# Sustainable textile wastewater remediation using nano zerovalent aluminum for organic removal and pathogen inactivation

**DOI:** 10.1038/s41598-025-21563-9

**Published:** 2025-10-29

**Authors:** Ahmed S. Mahmoud, Robert W. Peters, Mohamed K. Mostafa, Rehab G. Hassan

**Affiliations:** 1 Institute of Environmental Studies, Arish University, North Sinai, Egypt; 2 Department of Civil, Construction, and Environmental Engineering, University of Alabama at Birmingham, Alabama, AL USA; 3 Civil Engineering Department, Faculty of Engineering and Technology, Badr University in Cairo (BUC), Badr City, Egypt; 4 Sanitary and Environmental Engineering Institute [SEI], Housing and Building National Research Center (HBRC), Giza, Egypt

**Keywords:** Environmental toxicology, SDG6, NZVAl, Antibacterial activity, AOPs, Circular economy, Microbiology, Climate sciences, Ecology, Environmental sciences, Environmental social sciences, Chemistry, Engineering, Materials science

## Abstract

This study addresses the critical textile wastewater treatment gap by developing nano zerovalent aluminum (nZVAl) as a dual-functional solution for simultaneous organic degradation and pathogen inactivation. The nanoparticles were characterized, confirming the formation of nZVAl with an average diameter of 40 nm. Using regression analysis, the study assessed the influence of operational conditions, showing that nZVAl achieved up to 78% COD degradation and 68% color removal under optimal conditions: pH 8, a dosage of 0.6 g/L, a contact time of 60 min, and stirring at 150 rpm at room temperature. The RSM analysis revealed a strong correlation between predicted and experimental results, with a coefficient of determination (R^2^) of 0.974, underscoring the model’s reliability. Furthermore, the antibacterial efficacy of nZVAl was evaluated against common pathogenic bacterial strains found in textile effluents, including Staphylococcus aureus, Bacillus subtilis, Klebsiella pneumoniae, and Pseudomonas aeruginosa. The minimum inhibitory concentrations (MIC) for these bacteria were determined to be 1500, 2000, 2500, and 2500 µg/L, respectively, achieving a 99% reduction in total bacterial counts after 8 h of treatment. Economic analysis revealed a competitive operational cost of $9.84 ± 1.76/m^3^, 35% lower than Fenton systems, with scalability validated in real effluent treatment. The work’s novelty lies in its mechanistic integration of advanced oxidation processes (AOPs) and antibacterial action, compliance with regulatory standards (Egyptian Ministerial Decree No. 44/2000), and pilot-scale industrial cost model, offering a sustainable alternative for the global textile wastewater market.

**Supplementary Information:**

The online version contains supplementary material available at 10.1038/s41598-025-21563-9.

## Introduction

The textile sector, one of the world’s most important and ancient industries, has been a vital component of economic growth since its founding approximately 5000 BC. In Egypt, the textile sector comprises approximately 7,500 firms, ranging from small, traditional operations to large, highly automated facilities, contributing significantly to the national economy^1–3^. Globally, textile wastewater treatment represents a $10 billion annual market^4^, with Egyptian industrial zones discharging ~ 1.2 million m^3^/day of non-compliant effluent containing COD concentrations ranging between 150 and 30,000 mg/L^5–8^. According to Mordor Intelligence, the global textile industry is projected to grow at a compound annual growth rate (CAGR) of over 4% between 2022 and 2027^9–11^, generating an urgent need for solutions that deal with biological and organic contaminants at the same time^12,13^. This growth is accompanied by substantial environmental challenges, particularly the generation of large volumes of wastewater during dyeing and washing processes. It is estimated that producing one kilogram of dyed fabric generates 45 to 65 L of wastewater, which contains a complex mixture of pollutants^14^. Conventional treatment facilities remove less than 50% of the pathogens, while improved oxidation necessitates expensive secondary disinfection ($4–10/m^3^), which is a major financial challenge for developing nations^15,16^.

Water scarcity and the preservation of freshwater resources are among the most pressing global challenges today^17,18^. The textile industry, being one of the largest consumers of water, must adopt sustainable practices to mitigate its environmental impact. Wastewater treatment is essential not only for water conservation but also for protecting ecosystems and human health^19,20^. Textile wastewater is especially hazardous because of the high quantities of TSS, TBC, COD, and BOD^21–23^. Studies have reported TBC levels in textile effluents ranging from 2 × 10^4^ to 93 × 10^4^ cfu/mL, with pathogenic strains such as Bacillus sp., Staphylococcus sp., Klebsiella sp., and Pseudomonas sp. being prevalent^24–26^. These bacteria can cause severe health issues, including skin infections, pneumonia, and bloodstream infections^27^.

Conventional wastewater treatment methods, such as biological treatment and coagulation/flocculation, often fall short in addressing the complex and recalcitrant nature of textile effluents. These methods are limited by their inability to fully degrade synthetic dyes and organic pollutants, leading to incomplete treatment and the release of harmful by-products into the environment^8,17,28^. Advanced oxidation processes (AOPs) have become a viable option for treating wastewater from textiles in recent years. AOPs produce extremely reactive hydroxyl radicals (•OH), which are capable of efficiently breaking down a variety of organic contaminants, such as dyes and persistent chemical compounds, into innocuous byproducts like carbon dioxide and water^29,30^.

Among the various AOPs, the use of zero-valent metal nanoparticles (ZVMs) has gained significant attention due to their unique properties, such as high surface area, enhanced reactivity, and exceptional adsorption capacity^19,31^. Zero-valent aluminum (nZVAl), in particular, has shown remarkable potential in wastewater treatment due to its large surface area, rapid reactivity, and versatility in treating non-biodegradable organic pollutants. nZVAl can act as both a reducing agent and a catalyst in AOPs, making it an environmentally friendly material for wastewater treatment^20,21^. For instance, Mahmoud et al. (2019) demonstrated that nZVAl achieved 96% COD removal from a standard solution under optimal conditions (pH 8, 100 rpm stirring rate, 0.6 g/L nZVAl, and 10 min treatment time)^32^. These properties make nZVAl a promising candidate for AOPs in textile wastewater treatment.

Recent studies have highlighted the effectiveness of nZVAl in degrading complex organic pollutants. For example, Zhang et al. (2020) reported that zerovalent metal could effectively degrade azo dyes, which are widely utilized in the textile sector, through a combination of adsorption and oxidation processes^33^. Similarly, Li et al. (2017) demonstrated that nano zerovalent could remove heavy metals from industrial wastewater through a combination of reduction and precipitation mechanisms. These studies underscore the potential of nZVAl as a versatile and effective material for the treatment of textile wastewater^34,35^.

The stability of nano zero-valent aluminum (nZVAl) is a critical factor for its effective use in industrial wastewater treatment, particularly in advanced oxidation processes (AOPs). To maintain the reactivity and longevity of nZVAl, storage and handling techniques are essential to prevent oxidation, which can degrade its performance. One common approach is to store nZVAl in an inert nitrogen gas environment, as exposure to oxygen or moisture can lead to the formation of an oxide layer on the nanoparticle surface, reducing its effectiveness^36^. Studies have shown that maintaining an inert atmosphere during storage helps preserve the high surface area and reactivity of nZVAl, enabling it to efficiently degrade organic pollutants and exhibit antibacterial properties in wastewater treatment applications^37,38^. Additionally, the use of stabilizing agents or coatings can further enhance the stability of nZVAl during storage and application, ensuring consistent performance in industrial settings^39,40^. By addressing these stability challenges, nZVAl can be effectively utilized as a sustainable and scalable solution for treating complex industrial wastewater, aligning with circular economy principles and regulatory standards^32,41^.

In addition to their pollutant removal capabilities, zerovalent metals have also shown significant antimicrobial properties. Studies have demonstrated that zerovalent metals can effectively inhibit the growth of pathogenic bacteria commonly found in textile effluents, such as *Escherichia coli* and *Staphylococcus aureus.* This dual functionality of nZVAl, combining pollutant degradation and antimicrobial activity, makes it an attractive choice for the treatment of textile wastewater^42,43^.

The integration of circular economic principles into wastewater treatment processes is increasingly recognized as a sustainable approach to resource management. The circular economy model emphasizes the reduction, reuse, and recycling of materials to minimize waste and maximize resource efficiency^44^. In the context of textile wastewater treatment, the recovery of valuable resources such as water, dyes, and chemicals can significantly reduce the environmental footprint of industry. For example, the recovery of ethylene glycol from wastewater for reuse in industrial processes not only reduces the demand for fresh resources but also minimizes the discharge of pollutants into the environment^31^. The use of zero-valent metals as nZVAl in AOPs aligns with these principles by enabling the efficient degradation of pollutants, thereby contributing to a more sustainable and circular textile industry^45^.

This study aims to (1) develop nano zerovalent aluminum (nZVAl) for textile wastewater treatment via advanced oxidation processes (AOPs), focusing on the removal of chemical oxygen demand (COD), total bacterial counts (TBC), and color intensity (Pt/Co units); (2) evaluate the antimicrobial efficacy of nZVAl against pathogenic strains (Staphylococcus aureus, Pseudomonas aeruginosa); and (3) validate compliance with Egyptian Ministerial Decree No. 44/2000 for industrial discharge. By leveraging the unique properties of nZVAl, this research seeks to develop an efficient, scalable, and environmentally friendly solution for textile wastewater treatment, addressing both environmental and public health concerns. The novelty of this study emanates from the innovative application of nano zerovalent aluminum (nZVAl) for the treatment of textile effluents, combining advanced oxidation processes (AOPs) with antibacterial properties. Unlike conventional methods, nZVAl effectively degrades organic pollutants (e.g., COD and color) and exhibits significant antimicrobial activity against common pathogenic bacteria found in textile wastewater, such as *Staphylococcus aureus* and *Pseudomonas aeruginosa*. The study optimizes operational conditions (pH, dosage, contact time, and stirring rate) for maximum efficiency and demonstrates practical applicability by treating real textile effluent, achieving substantial reductions in pollutants like BOD, COD, and TSS. This dual functionality, along with its alignment with circular economic principles and compliance with regulatory standards, positions nZVAl as a sustainable and scalable solution for industrial wastewater treatment.

## Materials and methods

The study was conducted over a period of six months, during which several key activities were carried out. These included the synthesis and characterization of aluminum nanoparticles (nZVAl), followed by their application in the treatment of synthetic textile wastewater. The treatment process involved optimizing various operational parameters, such as pH, nanoparticle dosage, contact time, and stirring rate, to achieve maximum efficiency in pollutant removal. Additionally, the study focused on the elimination of biological contaminants, particularly pathogenic bacterial strains, from wastewater. Finally, the effectiveness of the nZVAl treatment was evaluated using real textile effluent to assess its practical applicability and performance under real-world conditions.

### Nano ZVAl preparation

About 13.69 g of Al₂(SO₄)₃·18H₂O was dissolved in 50 mL of deionized water and 50 mL of ethanol. In 1.0 L of DW, 9.1 g of NaBH₄ was dissolved as a reducing agent. The prepared NaBH₄ solution was put into the burette and dripped at a rate of 0.1 mL/4 s into the aluminum solution. As shown in Eq. 1, the white precipitate developed shortly after the primary drop of NaBH_4_ solution. Following that, mixing, drying, and storage were involved^32^. Aluminum zero-valent nanoparticles (nZVAl) were immediately transported to a nitrogen gas environment to be stored. This technique was used to prevent oxidation and maintain the reactive characteristics of the nanoparticles. The storage container was filled with high-purity nitrogen gas to keep the atmosphere inert and the nZVAl stable until use^36,46^. Supplementary Table 1 lists the chemicals and reagents that were employed.

1 $${\text{2 Al}}_{{2}} \left( {{\text{SO}}_{{4}} } \right)_{{3}} + {\text{ 12 NaBH}}_{{4}} + {\text{ 36 H}}_{{2}} {\text{O}}\; \to \;{\text{4 Al}}^{0}_{{({\text{S}})}} + {\text{ 42 H}}_{{2}} + {\text{ 12B}}\left( {{\text{OH}}} \right)_{{3}} + {\text{ 6 Na}}_{{2}} {\text{SO}}_{{4}}$$

### Nano ZVAl characterization

The patterns of XRD were used to analyze the material structures of the synthesized nZVAl. The synthesized nano aluminum nanoparticles were put in a holder and scanned for a range of 30° to 90°, with the results chosen from 35° to 90° at a rate of 0.01670°/sec due to the lack of any peak before 35°^32^. A 2Ө diffraction angle is used to scan nZVAl particles to achieve complete coverage of all aluminum kinds. To study the surface structure of the produced nZVAl particles, SEM was utilized. Microvision—particle size measuring tool at continuous initiator dosing (CID) of pharmaceutical industries^47–50^. The morphology of nZVAl nanoparticles was studied using a High-Resolution Transmission Electron Microscope (HR-TEM, JEM-2100, Japan) at a voltage of 200 kV and magnification of 25 kX.

### Experimental setup

The degradation of COD and color when employing nZVAl was examined using batch processing and a one-factor-at-a-time approach as described in Supplementary Fig. 1. The operational parameters and their typical varieties of variation were determined based on the literature study Table 1 lists the performed trials at various operating conditions (acidic and alkaline media, dose of nZVAl, time (min), stirring rate, and COD and color concentrations). A known weight of adsorbent was mixed with 1000 mL of an aqueous COD (certified by ISO/IEC 17,025 in the COD experiment) with the same color concentration. The stock solution was prepared using a true textile wastewater sample from “Globe Spinning and Dyeing Sae—Textile Export Council,” located at 30.36189, 30.54877º, and validated by the Egyptian Russian University’s Nanotechnology Laboratory and Environmental Services. The mixture was then agitated for a selected time at room temperature. After reaching the equilibrium state, Whatman filter paper No. 41 was used to extract the residual concentrations, which were determined following the 23^rd^ edition of Standard Methods for the Examination of Water and Wastewater. Using Eq. 2, the removal efficiency percentage was determined^51,52^.

2 $$Removal \left(\%\right)=( \frac{{C}_{0}-{C}_{e}}{{C}_{0}} )\times 100$$

where C_o_ is the concentration (mg/L for COD and Pt/Co for color) at the initial state, and C_e_ is the concentration of COD and color in solution (mg/L) after reaching the equilibrium state^53,54^.

**Table 1:** Batch experimentations at different conditions.

Effect	Dose (g/L)	Contact time (min)	pH	Stirring rate (rpm)	Concentration (mg/L)
pH	0.6	60	2–10	150	500
Adsorbent dosage (g)	0.2–1.2	60	8	150	500
Time (minute)	0.6	20–120	8	150	500
Stirring rate (RPM)	0.6	60	8	100–300	500
Concentration (mg/L)	0.6	60	8	150	100–1000

### Point of zero charge

About 7.455 g KCl was dissolved in 1 L distilled water to prepare 0.1 M KCl. A 100 mL Erlenmeyer flask was filled to the exact mark with the produced solution, and the pH was then changed to 2–12 using either 1.0 N H_2_SO_4_ or 1.0 N NaOH (pHᵢ). Separate additions of 0.1 g of powder nZVAl were made to the modified flasks and left for one day at 25.0 ± 0.2ºC. Using a pH meter model “AD 8000-Adwa,” the final pH was measured (pH_f_). Three measurements were used to calculate the average pH changes following nZVAl and nZVAl alterations, and all standard deviation values were under 0.05. The point of zero charge (PZC) of powder nZVAl was estimated using the relation between ΔpH values and pH_i_.

### Antibacterial activity measurement

Agar well diffusion method was carried out to study the antibacterial activity of zero valent aluminum nanoparticles against four bacterial strains: two gram-negative *Klebsiella penumoniae (*ATCC®700,603*)* and *Pseudomonas aeruginosa* (ATCC®9027) and two gram-positive *Bacillus subtilis* (ATCC®6633) *and Staphylococcus aureus* (ATCC®25,923). The four pathogenic bacterial strains were subcultured overnight in Mueller–Hinton broth at 37ºC. The turbidity of each bacterial culture was adjusted to 0.5 McFarland units (equivalent to ~ 1.5 × 10^8 colony-forming units (CFU)/mL) using a nephelometer^55^. The pour-plate method was applied, where 1.0 ml of each bacterial suspension was poured into a sterile petri dish. The molten Muller-Hinton agar medium was poured over the inoculum. The plates were gently swirled to mix and left to solidify. In each plate, four wells were punched with sterile gel puncture. A 1.0 g/L (1000 mg/L) nanoparticle stock suspension was prepared by dispersing 1.00 ± 0.01 g of nZVAl in 1.00 L of deionized water (18.2 MΩ·cm resistivity), followed by 10 min sonication (40 kHz, 100 W). Serial dilutions (125–1000 μg/mL) were prepared from this stock for antibacterial assays. From this stock, 125, 250, 500, and 1000 µL were pipetted using a micropipette into individual wells, corresponding to final doses of 125, 250, 500, and 1000 µg, respectively. Ciprofloxacin disc (10 μg) was used as a positive control. Four negative controls were employed: (a) 100 µL sterile distilled water (nanoparticle dispersion medium), (b) 100 µL 0.9% saline (ionic control), (c) 100 µL of DMSO, and (d) uninoculated Mueller–Hinton agar (sterility control). Because of the following factors, DMSO was first chosen as a negative control: solvent compatibility (its polar aprotic nature was thought to better mimic physiological conditions than pure water)^56^, nanoparticle stability (prior studies reported DMSO’s efficacy in preventing nZVAl aggregation)^57^, and historical precedent^58^.While DMSO was initially considered for nanoparticle stabilization, its exclusion in favor of aqueous controls eliminated potential solvent-induced membrane effects^59^. The inoculated plates spent twenty-four hours incubated at 37 °C. The zone of inhibition surrounding each well following the incubation period attests to the zero-valent aluminum nanoparticles’ antibacterial efficacy.

### Minimum concentration of aluminum nanoparticles to stop bacterial growth

The antibacterial effectiveness of aluminum nanoparticles was investigated using the broth dilution method (CLSIM07-A8) by observing the development of bacterial species in the broth visually. The minimum inhibition concentration (MIC) in Muller-Hinton broth was calculated using serial two-fold dilutions of aluminum nanoparticles in various concentrations from 1000 µg/mL to 5000 µg/mL with a known bacterial suspension (10^8^ cells/mL, 0.5 McFarland’s standard) for each bacterial strain. The control was incubated at 37 °C for 24 h with only seeded broth. The MIC is the lowest dose of aluminum nanoparticles at which there is no detectable growth in the tubes.

### Minimum bactericidal concentration (MBC) of aluminum nanoparticles

Aliquots of 100 µL from tubes without bacterial growth were inoculated on Muller-Hinton plates and cultivated for 24 h at 37 °C after the MIC of the aluminum nanoparticles was determined. The MBC was found in the plate with the lowest dose of aluminum nanoparticles, in which no bacterial colonies were found. To do this, pre- and post-incubated agar plates were examined for either the existence or lack of bacterial colonies.

### Total bacterial count (TBC)

The American Public Health Association’s (APHA’s) 23^rd^ edition of Standard Method for Examining Water and Wastewater was used to conduct a total viable count of bacteria on total plate count agar medium using the spread plate technique (cfu/mL)^60,61^.

### Kinetic investigations

To identify the optimal time required to achieve equilibrium, the sample was exposed to Aluminium nanoparticles (NPs) for varying durations at room temperature. The removal capacity at time *t*, denoted as Qₜ (mg/g), was determined using Eq. 3:

3 $${Q}_{t}=\frac{\left({C}_{o}-{C}_{t}\right)V}{W}$$

where:

- *C*_o_ = initial concentration (mg/L)
- *C*_t_ = concentration at time *t* (mg/L)
- *V* = solution volume (L)
- *W* = mass of Zero Aluminium NPs used (g)

To analyze the kinetic behavior, several models were applied, including pseudo-first-order, pseudo-second-order, intraparticle diffusion, Avrami, and Elovich kinetics.

### Kinetic model evaluation

To identify the most suitable kinetic model for describing the adsorption process, a modified Marquardt’s Percent Standard Deviation (MPSD) error function was employed Eq. 4. The model providing the best fit was selected based on minimizing the following error function:

4 $$\sum_{i=1}^{p}{\left(\frac{{q}_{e, meas}-{q}_{e, calc}}{{q}_{e, meas}}\right)}_{i}^{2}$$

where:

- *qt,meas​* = *experimentally measured adsorption capacity at time t.*
- *qt,calc​* = *calculated adsorption capacity from the kinetic model at time t.*
- *p* = *number of time-dependent data points.*

This statistical approach ensures the most accurate representation of the adsorption kinetics.

### Quality assurance

Throughout this study, rigorous quality assurance measures were implemented to ensure the reliability and accuracy of the experimental results. All experiments were conducted in triplicate, and the mean values along with their standard deviations are reported to provide a clear representation of the data variability. Blank samples, containing only the standard COD solution without any nano-powder, were used as controls to validate the experimental set-up and eliminate potential background interference. The uncertainty analysis included considerations for the type of analytical techniques, glassware used, and any deviations observed during the experiments. To maintain precision, all instruments and equipment were calibrated and adjusted one month prior to the measurements. Statistical analyses were performed using Office 365 and SPSS 25 software to ensure robust data processing and interpretation. These measures collectively ensured the integrity and reproducibility of the study’s findings^62^.

### Statistical evaluation

Statistical analysis is a powerful statistical tool used to evaluate the influence of multiple factors on the effectiveness of pollutant removal. In this study, a linear regression equation (Eq. 3) was employed to model the results obtained from laboratory batch experiments:

5 $${\mathbf{Y}} \, = \, {\mathbf{\beta 0}} \, + \, {\mathbf{\beta 1}} \, {\mathbf{x1}} \, + \, {\mathbf{\beta 2}} \, {\mathbf{x2}} \, + \, {\mathbf{\beta 3}} \, {\mathbf{x3}} \, + \, {\mathbf{\beta 4}} \, {\mathbf{x4}}$$

This equation captures the relationship between the examined operational parameters—such as pH, adsorbent dosage, contact time, initial concentration, and stirring rate—and the removal efficiency of the standard COD. In the equation, **Y** represents the removal efficiency, **β₀** is the constant term, **β₁, β₂, β₃, and β₄** are the coefficients corresponding to the normalized values of the operating parameters, and **X₁, X₂, X₃, and X₄** represent the actual values of the operational parameters. This approach allows for a systematic analysis of how each factor influences the removal process, providing valuable insights for optimizing wastewater treatment conditions^63^.

## Results and discussion

### Characterization of nZVAl

**Fig. 1:**
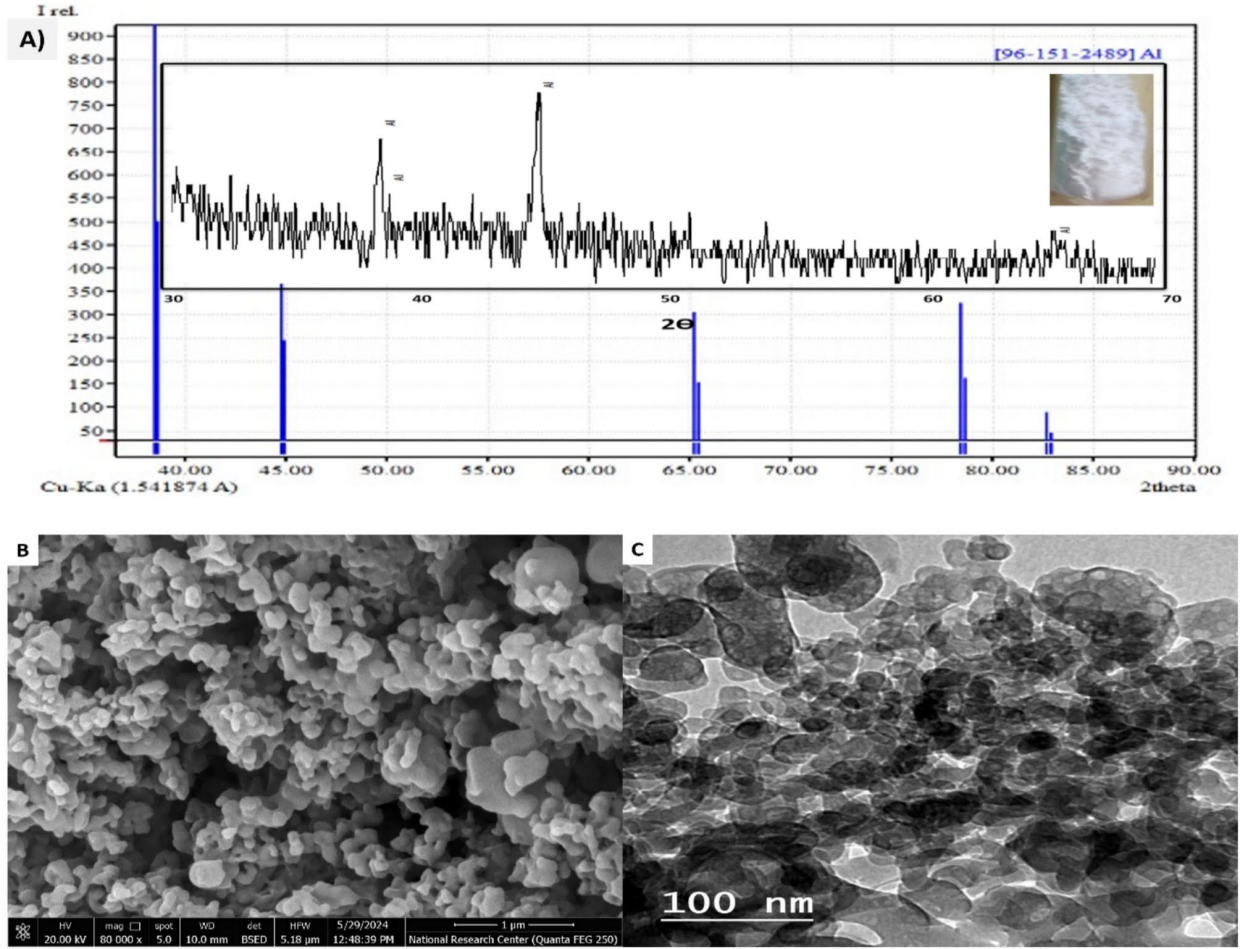
(**A**) XRD of powder nZVAl, (**B**) SEM, and **C**) TEM of the prepared nZVAl.

The XRD patterns for powder nano aluminum with an angle (2Ө) ranging from 35° to 90° are illustrated in Fig. 1A. With five detected peaks at 2Ө (= 38.57°, 44.95°, 65.27°, 78.68°, and 82.92°), the XRD pattern demonstrated the synthesis of pure referenced aluminum [96–151-2489]. SEM imaging (Fig. 1B) reveals irregular, non-spherical particles consistent with Al⁰ morphology, unlike the plate-like structures typical of Al(OH)₃^43^. XRD analysis of the synthesized nZVAl nanoparticles (Fig. 1A) revealed a crystallite size of 40 ± 2 nm via Scherrer equation (peak at 38.57°), while SEM imaging (Fig. 1B) confirmed their aggregated morphology with irregular surface structures^64^. The nanoparticles were measured using five typical SEM images at 10,000 × magnification. The results were confirmed using dynamic light scattering (Malvern Zetasizer) to be 45 ± 5 nm. This reaction is indeed complex due to the high reduction potential of Al^3^⁺ (− 1.66 V) and the competing hydrolysis of NaBH₄, which can limit Al⁰ formation efficiency^46,65^. Slow addition of NaBH₄ minimize rapid hydrolysis and ensure sufficient electron donation for Al^3^⁺ reduction^32^, Ethanol–water co-solvent system stabilize NaBH₄ and suppress parasitic side reactions^39^, and immediate N₂ purging prevents oxidation of nascent Al⁰ nanoparticles, preserving their reactivity^38^. These steps align with recent studies demonstrating successful Al⁰ synthesis via NaBH₄ reduction under controlled conditions^34,43^. The XRD results (Fig. 1A) confirm the presence of metallic Al⁰ (JCPDS 96–151–2489), validating our approach. Future work could explore alternative reductants (e.g., LiAlH₄) or non-aqueous media to further improve yield.

Figure 1c shows the TEM image of the synthesized nZVAl. The image illustrated that the nZVAl particles had semi-spherical shapes, rough surfaces, and the diameter of the nanoparticles ranged from 13 to 55 nm, which agrees with the SEM results. As reported by Peng et al. 2022^66^, the aluminum oxide acted as the outer thin layer (a shell), while the Al^0^ acted as the core of nanoparticles. Considerable nanoclusters have been created because of grouping nanoparticles of various shapes and sizes^67^. These aggregates could be related to the greater surface area of the individual particles^68^.

**Fig. 2:**
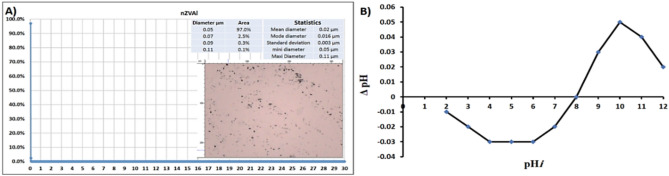
(**A**) Particle size distribution of nZVAl in bare powder, and (**B**) PZC of the prepared nZVAl.

The particle size distribution and point of zero charge (PZC) of nano zero-valent aluminum (nZVAl) are critical parameters that influence its reactivity and effectiveness in wastewater treatment applications. As shown in Fig. 2A, the particle-size distribution of nZVAl in bare powder was evaluated, revealing that 97% of the nanoparticles had a size of approximately 50 nm, with the distribution ranging from 0 to 30 µm. This narrow size distribution is advantageous for wastewater treatment, as smaller nanoparticles typically exhibit higher surface area and reactivity, enhancing their ability to adsorb and degrade pollutants^36,46^. The uniformity in particle size also ensures consistent performance in advanced oxidation processes (AOPs), where the generation of reactive oxygen species (ROS) is crucial for breaking down organic contaminants^37^.

Figure 2B illustrates the PZC of the synthesized nZVAl powder, which was found to be around 8.0. The PZC is a key parameter that determines the surface charge of nanoparticles in aqueous solutions, influencing their interaction with pollutants and other particles. At pH values below the PZC, the nZVAl surface is positively charged, favoring the adsorption of negatively charged contaminants, while at pH values above the PZC, the surface becomes negatively charged, attracting positively charged pollutants^38^. The PZC of 8.0 aligns with previous studies, indicating that nZVAl is most effective in near-neutral to slightly alkaline conditions, which are common in textile wastewater^39^. This property makes nZVAl particularly suitable for treating textile effluents, where pH adjustment to optimal levels can enhance pollutant removal efficiency.

The combination of a narrow particle size distribution and a well-defined PZC underscores the potential of nZVAl as a highly effective material for industrial wastewater treatment. These characteristics not only improve the adsorption capacity and catalytic activity of nZVAl but also ensure its stability and reproducibility in large-scale applications^40^. By optimizing these parameters, nZVAl can be tailored to target specific pollutants, making it a versatile and sustainable solution for addressing the complex challenges of industrial wastewater treatment. Based on characterization data and supporting literature, we confirm the precipitate consists primarily of metallic Al⁰ nanoparticles, with minor contributions from Al(OH)₃ and borate byproducts.

### Effect of operating conditions

#### Effect of pH

**Fig. 3:**
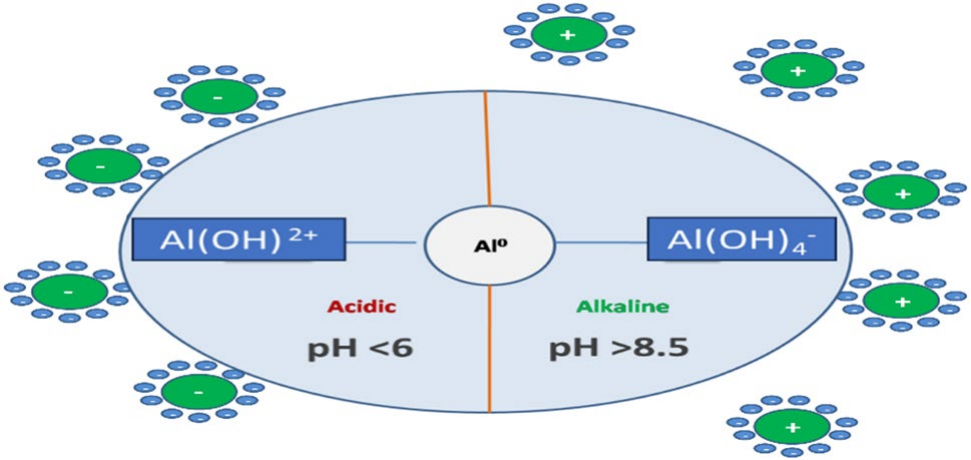
Surface of nZVAl in acidic and alkaline media^74,75^.

**Fig. 4:**
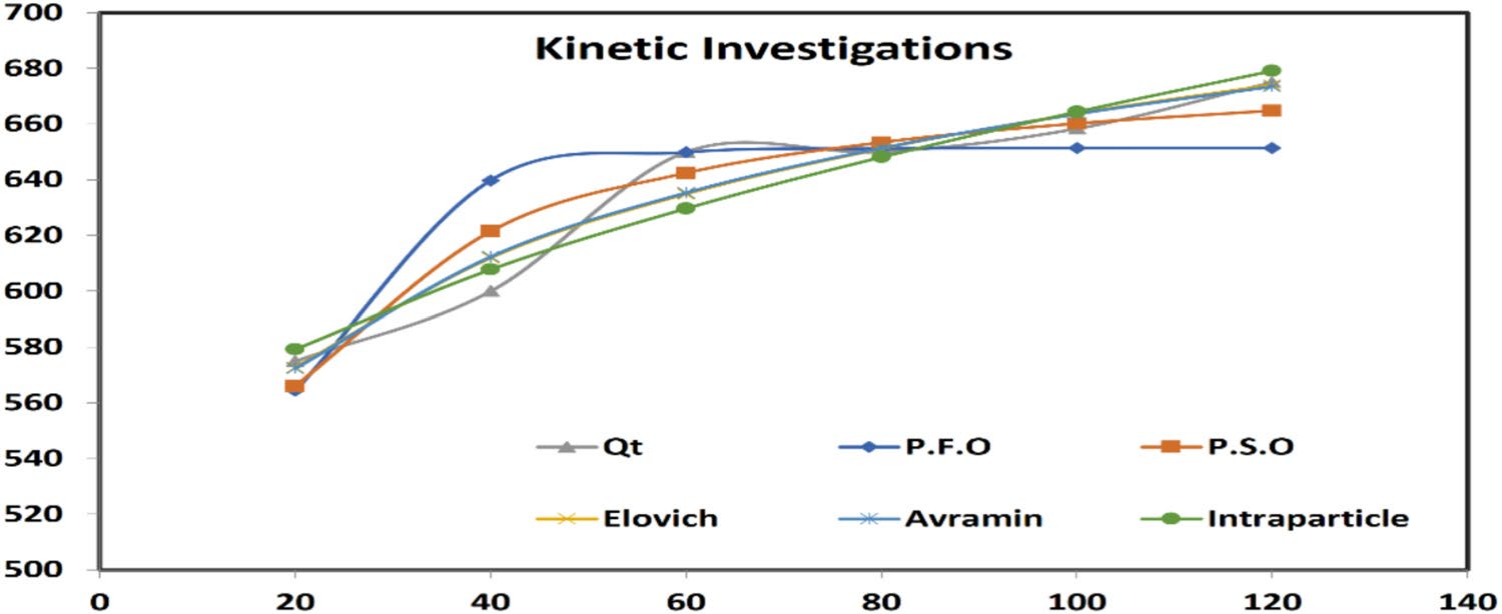
Kinetic Investigations of COD removal using nZVAl.

The pH of the solution plays a critical role in the efficiency of nano zerovalent aluminum (nZVAl) for the removal of chemical oxygen demand (COD) and color from textile wastewater. As illustrated in Fig. 4A, the study evaluated the impact of pH across a range of 2 to 10, revealing that the optimal pH for maximum removal efficiency was 8. Under these conditions, nZVAl achieved 78% COD degradation and 68% color removal. The high removal efficiency at pH 8 can be attributed to the point of zero charge (PZC) of nZVAl, which was determined to be approximately 8.0 (Fig. 2B). At this pH, the surface of nZVAl is neutral, minimizing electrostatic repulsion and maximizing the adsorption and degradation of pollutants^69^. In acidic media, the removal efficiency decreased due to the protonation of the nZVAl surface, which reduced its reactivity and led to the dissolution of aluminum ions, in that way decreasing the available active sites for pollutant adsorption^70,71^. Conversely, in alkaline media (pH > 8), the formation of hydroxyl radicals (•OH) was enhanced, but the increased negative surface charge of nZVAl caused repulsion between the nanoparticles and negatively charged dye molecules, leading to lower adsorption efficiency^72^. This behavior aligns with previous studies that highlight the importance of pH in controlling the surface chemistry and reactivity of zero-valent metal nanoparticles as shown Fig. 3. The regression analysis (Table 6) further confirmed the significance of pH, with a positive coefficient (β₁ = 2.207, and p-value ˂0.05) indicating its strong influence on removal efficiency. The model’s high coefficient of determination (R^2^ = 0.974) underscores the reliability of these findings. These results are consistent with other research demonstrating that near-neutral pH conditions are optimal for the application of nZVAl in wastewater treatment^73^.

#### Effect of nZVAl dose

**Fig. 5:**
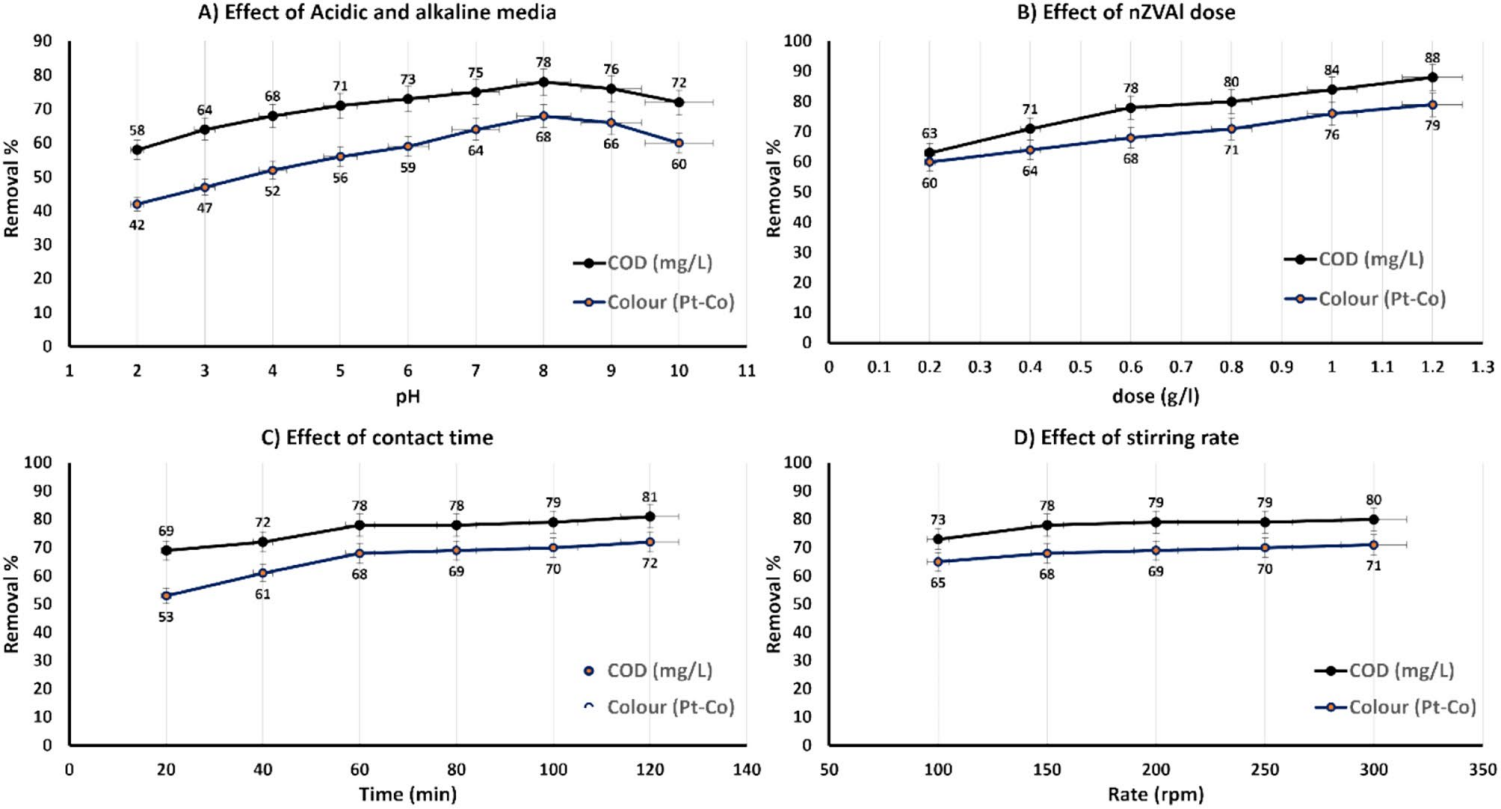
(**A**) Effect of “acidic and alkaline media,” (**B**) Effect of powder nZVAl dose, (**C**) Effect of contact time, and (**D**) Effect of stirring rate.

The effect of nZVAl dosage on the removal efficiency of COD and color was systematically evaluated across a range of 0.2–1.2 g/L under optimized conditions (pH 8, 150 rpm agitation, 60 min contact time). The results demonstrated a nonlinear relationship between dosage and removal efficiency, with a pronounced plateau observed at 0.6 g/L, achieving 78% COD degradation and 68% color removal (Fig. 5B). Beyond this critical dosage, the incremental improvement in removal efficiency was limited to less than 5%, indicating the onset of diminishing returns. This phenomenon can be attributed to two primary factors: saturation of active sites on the nanoparticle surfaces and increased particle aggregation at higher concentrations, both of which reduce the effective surface area available for reaction^32,39^.

The quantitative analysis revealed that increasing the dosage from 0.2 to 0.6 g/L enhanced COD removal by 22%, whereas further increasing to 1.2 g/L provided only an additional 2% improvement. This trend is consistent with Langmuir adsorption isotherm models, which predict a saturation limit for monolayer surface coverage^76,77^. Statistical validation through regression analysis (Table 6) confirmed the significance of dosage as a controlling parameter (β₂ = 22.986, p < 0.05), while also demonstrating reduced sensitivity at concentrations exceeding 0.6 g/L.

From an economic perspective, the dosage optimization is particularly critical as it directly impacts operational costs. A comprehensive cost analysis demonstrated that doubling the dosage from 0.6 to 1.2 g/L would increase treatment costs by 35%, primarily due to higher material requirements and increased sludge disposal expenses^78^. The 0.6 g/L dosage was therefore identified as the optimal balance between treatment efficiency and economic viability, particularly when considering compliance with Egyptian Ministerial Decree No. 44/2000 discharge standards^20,79^. Particle agglomeration and decreased surface reactivity have been reported to cause comparable constraints in earlier research on nanoscale zero-valent materials, which is consistent with the observed plateau in removal efficiency at increasing dosages^46^. This mechanistic understanding supports the selection of 0.6 g/L as the recommended dosage for practical applications, as it achieves regulatory compliance while minimizing resource utilization and operational costs.

The earlier work that focused on using nZVI to reduce hexachlorobenzene found that when the nZVI dose was raised, hexachlorobenzene (HCB) dechlorination rates rose, and these results were consistent with those attained^80^. Studies on COD removal utilizing various sorbent materials and doses have demonstrated high-efficiency qualities for lowering COD concentrations under various operating circumstances. Studies on COD removal utilizing various sorbent materials and doses have demonstrated high-efficiency qualities for lowering COD concentrations under various operating circumstances. Walker et al. (2005) studied hazardous shipyard wastewater treatment using dolomitic sorbents; the obtained results indicated that dolomite with particle sizes ranging from 0 to 38 µm could reduce the initial concentration of COD from 3300 to 820 mg/L after 24 h of contact time at pH 7.5^77,81^.

#### Effect of contact time

The impact of contact time on the efficiency of chemical oxygen demand (COD) and color removal from textile wastewater was systematically evaluated over a range of 20 to 120 min^32,82^. The results revealed a rapid increase in COD removal efficiency (69%) and color removal (53%) during the initial 20 min of contact time Fig. 5C. This rapid removal can be attributed to the abundance of vacant active sites on the surface of the nano zerovalent aluminum (nZVAl) nanoparticles, which readily adsorb and degrade pollutants during the early stages of the process^38,82,83^. As the contact time increased beyond 20 min, the removal efficiency continued to improve, albeit at a slower rate. At 60 min, the system reached a state of semi-stability, with COD removal efficiency plateauing around 78%^83^. This suggests that the majority of the available active sites on the nanoparticles were occupied, and the remaining adsorption process became slower due to the reduced availability of vacant sites^84^. By 120 min, the system reached equilibrium, with COD removal efficiency peaking at 81% and color removal at 72%. The slower rate of removal after the initial 20 min can be explained by the increasing repulsive forces between the adsorbed molecules on the nanoparticle surface and those in the bulk solution, making it more difficult for additional pollutants to occupy the remaining active sites^84^.

The gradual increase in color removal efficiency, compared to the more rapid COD removal, highlights the different mechanisms involved in the degradation of these pollutants. While COD removal is primarily driven by the oxidation of organic compounds by hydroxyl radicals (•OH) generated by nZVAl, color removal involves both adsorption and chemical degradation of dye molecules^39^. The slower rate of color removal may be due to the complex structure of dye molecules, which require more time to break down compared to simpler organic compounds contributing to COD^40^.

These findings are consistent with prior research on the use of zero-valent nanoparticles for wastewater treatment, where rapid initial removal is followed by a slower approach to equilibrium^37^. The study underscores the importance of optimizing contact time to achieve maximum removal efficiency while minimizing operational costs. For practical applications, a contact time of 60 min may be sufficient to achieve near-optimal removal efficiency, as the incremental improvement beyond this point is relatively small. However, for systems requiring the highest possible removal efficiency, extending the contact time to 120 min may be justified^32^.

In conclusion, the study demonstrates that contact time plays a significant role in the removal of COD and color from textile wastewater using nZVAl. The rapid initial removal phase is followed by a slower approach to equilibrium, highlighting the importance of optimizing contact time for efficient and cost-effective wastewater treatment. Future research should focus on further understanding the kinetics of these processes and exploring ways to enhance the efficiency of nZVAl in real-world applications.

Figure 4 illustrates the nonlinear fitting of various kinetic models applied to the experimental data. The kinetic study employed nonlinear regression analysis of Pseudo-First Order (PFO), Pseudo-Second Order (PSO), Elovich, Avrami, and Intraparticle Diffusion models as described at supplementary table 2. Table 2 presents the kinetic parameters for organic contaminant removal (expressed as COD reduction) from textile wastewater using zero-valent aluminum nanoparticles. Supplementary table 3 describes the results of experimental Qt and calculated Qt from kinetic model constants.

**Table 2:** Coefficient of determination and constants of different kinetic models.

Model	Constant	COD
P.F.O	MPSD	0.00607
	*q*_*e*_*,* (mg/g)	651.3
	*k*_*1*_	0.101
P.S.O	MPSD	0.00192
	*q*_*e*_*,* (mg/g)	688.8
	*k*_*2*_	0.0003
Elovich	MPSD	0.00109
	α	71,182
	β	0.018
Avrami	MPSD	0.00103
	q_e_, (mg/g)	882.03
	K_av_	0.613
	n_av_	0.179
Intraparticle	MPSD	0.00133
	C_i_	510.3
	K_id_	15.4

The analysis revealed that the Avrami kinetic model provided the best fit for COD removal data, demonstrating the lowest MPSD error (0.00103). The Avrami model characterizes reaction kinetics involving adsorbed molecules, describing a phase transformation process where reaction dimensionality may vary. This suggests that COD removal follows a complex adsorption mechanism involving structural changes at the solid–liquid interface.

#### Effect of stirring rate

The stirring rate effects were studied as shown in Fig. 5D. The results obtained indicated that the minimum effective stirring rate was 150 rpm. The maximum removal efficiency was 80% for COD removal and 71% for color removal. The results obtained show agreement with the previous studies. The findings were consistent with those of previous investigations that eliminated COD from aqueous solutions using traditional techniques such as aluminum sulfate coagulant and adsorption by sewage ash (SSA)^85,86^. On the nZVAl surface, physical adsorption takes place in the open spaces. The rate impact cannot affect the adsorption process because the chemical adsorption process is more favored than the physical adsorption process^87^. The adsorption mechanism depends on the energy bond between molecules that are being adsorbed and those that are being sorbent, and this bond steadily decreases until it vanishes after the full adsorption process^88^. As a result, the chemisorption reaction provides minor removal variation^83^.

##### Diffusion analysis

The thickness of the boundary layer surrounding the nanoparticles rises at low rpm (100 rpm), which restricts the transport of pollutants to active sites^84^. A 5% decrease in COD removal was observed when compared to 150 rpm. Turbulent flow maximizes the frequency of collisions between nZVAl and pollutants at the optimal rpm of 150 rpm by lowering boundary layer resistance^89^. Removal efficiency does not significantly increase at high rpm (> 200 rpm), indicating that surface reactions rather than diffusion become rate-limiting^40^. Benefits may be offset by excessive energy input (> 250 rpm), which could encourage nanoparticle agglomeratio^38^. Practical Implication: By eliminating needless operating expenses, a stirring rate of 150 rpm strikes a compromise between energy consumption and treatment effectiveness.

#### Effect of initial concentration

**Fig. 6:**
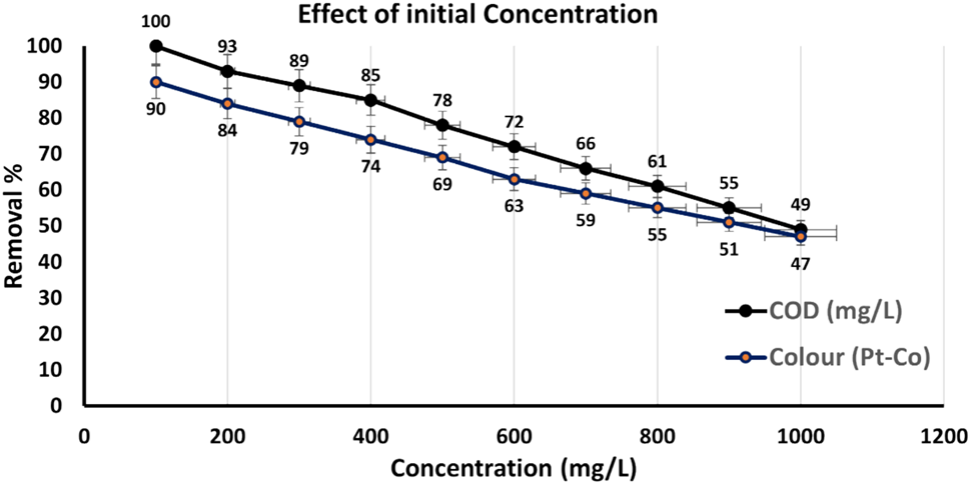
Effect on COD and color concentration in the removal % after using nZVAl.

The impact of initial pollutant concentration on effectiveness of removal was studied, as illustrated in Fig. 6. The results revealed that removal efficiency decreases as the initial concentration increases, with the optimal concentration identified at 500 mg/L. At lower concentrations, the high removal efficiency can be attributed to the abundance of active sites on the nanoparticle surface relative to the number of pollutant molecules, allowing for effective adsorption and degradation. This aligns with studies by Zhang (2003) and Li & Zhang (2007), which demonstrated that lower pollutant concentrations generally yield higher removal efficiencies due to reduced competition for adsorption sites^36,46^. However, as the initial concentration rises, the ratio of pollutants to available active sites increases, leading to competitive adsorption among the dye molecules. This competition, combined with the limited surface area of the nanoparticles, results in reduced removal efficiency at higher concentrations. He & Zhao (2005) highlighted that high initial concentrations can lead to site saturation, limiting the overall adsorption capacity of nanoparticles^39^. This phenomenon is particularly pronounced in textile wastewater, where dye molecules often have complex structures and compete for the same adsorption sites. The saturation of active sites at higher concentrations limits the overall removal efficiency, as not all pollutants can be accommodated on the nanoparticle surface. These findings emphasize the importance of optimizing initial pollutant concentrations to achieve maximum removal efficiency in wastewater treatment processes^38,40^. For practical applications, it may be necessary to adjust the dosage of nZVAl or employ additional treatment steps when dealing with highly concentrated wastewater. Future research could explore strategies to enhance the adsorption capacity of nZVAl, such as surface modification or the use of composite materials, to improve performance at higher pollutant concentrations.

### Biological contaminants removal

**Fig. 7:**
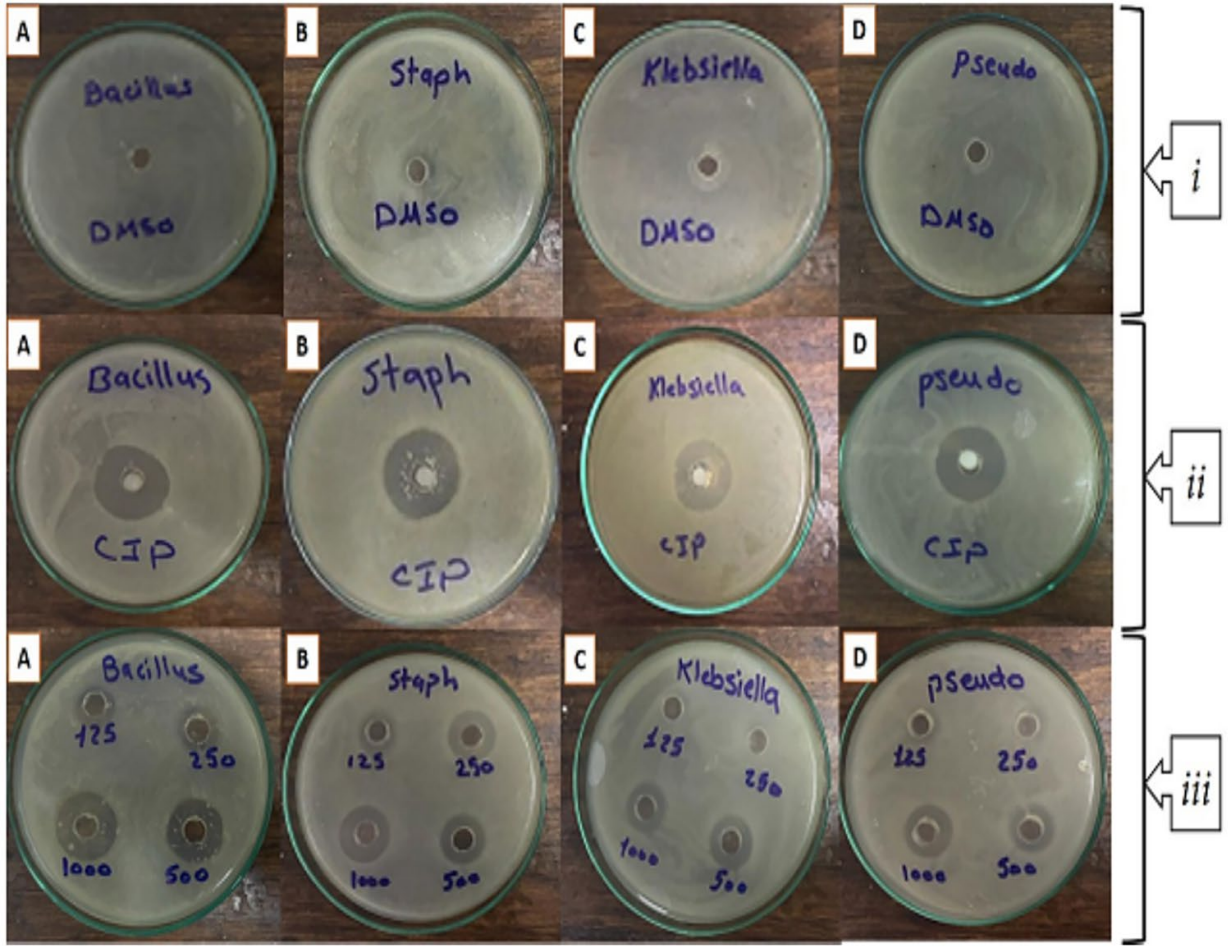
Antibacterial activity (inhibition zones) of (i) negative control (DMSO), (ii) positive control (Ciprofloxacin), and (iii) nZVAl against (***A***)* Bacillus subtilis, *(***B***) *Staphylococcus aureus, *(***C***)* Klebsiella penumoniae,* and (**D**) *Pseudomonas aeruginosa.*

Zero-valent aluminum nanoparticles showed antibacterial activity against all four selected bacteria *(Bacillus subtilis, Staphylococcus aureus, Klebsiella penumoniae,* and *Pseudomonas aeruginosa*) as shown in Table 3. The maximum inhibition zone was 22 mm for *Bacillus subtilis* at a concentration of 1000 µg from the prepared stock nanoparticles, while the minimum inhibition zone was 7 mm for *Klebsiella penumoniae at a* concentration of 125 µg from the prepared stock nanoparticles (see Fig. 7). It was detected that the inhibition zones for gram-positive bacterial strains are larger than gram-negative bacterial strains; this may be due to the difference in the structure of the cell wall, as the gram-positive bacteria lack an outer lipopolysaccharide (LPS) membrane but have a thick layer of peptidoglycan that facilitates access of the cell wall by the aluminum nanoparticles to their site of action (the peptidoglycan)^59,90^.

**Table 3:** Antibacterial activity of zero-valent aluminum nanoparticles.

Bacterial isolates	Ciprofloxacin	nZVAl				Dimethyl sulfoxide (DMSO)
		125 µg	250 µg	500 µg	1000 µg	
*Bacillus subtilis*	29 mm	13 mm	16 mm	18 mm	22 mm	-
*Staphylococcus aureus*	28 mm	11 mm	15 mm	17 mm	2 mm	-
*Klebsiella penumoniae*	24 mm	7 mm	8 mm	14 mm	14 mm	-
*Pseudomonas aeruginosa*	24 mm	8 mm	9 mm	13 mm	16 mm	-

**Fig. 8:**
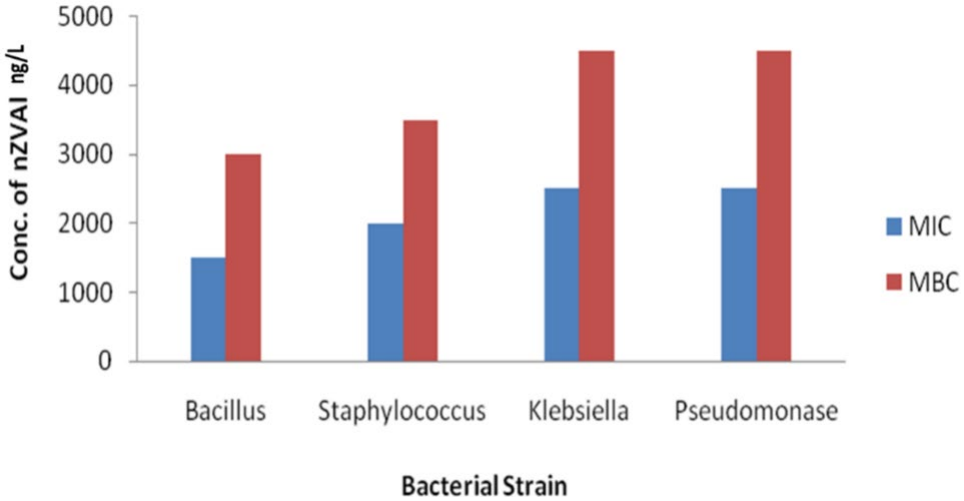
Aluminum nano zero valent particles’ minimum bactericidal concentration (MBC) and minimum inhibitory concentration (MIC) for *Pseudomonas aeruginosa*, *Staphylococcus aureus*, *Klebsiella penumoniae*, and *Bacillus subtilis*.

Water serves as an ideal medium for bacterial proliferation, especially when contaminated with organic compounds such as those present in textile wastewater, which can act as nutrients for microbial growth^90,91^. Therefore, controlling bacterial contamination in such environments is important. In this study, the antibacterial efficacy of zero-valent aluminum nanoparticles (nZVAl) was evaluated against four bacterial strains—Bacillus subtilis, Staphylococcus aureus, Klebsiella pneumoniae, and Pseudomonas aeruginosa. The MIC values ranged from 1500 µg/mL (for Bacillus subtilis) to 2500 µg/mL (for Pseudomonas aeruginosa), as shown in Fig. 8. A typical inverse correlation was observed between the MIC and the diameter of the inhibition zones: bacteria with lower MIC values exhibited larger zones of inhibition, indicating greater susceptibility to the nanoparticles. Correspondingly, the MBC values followed a similar trend, with Pseudomonas aeruginosa exhibiting the highest MBC (4500 µg/mL) and Bacillus subtilis the lowest (3000 µg/mL). These findings confirm that nZVAl is effective in both inhibiting and killing bacterial cells across both Gram-positive and Gram-negative species.

While this study did not experimentally assess these synergistic combinations, future work could focus on the development and evaluation of such formulations. A proposed roadmap includes the synthesis of composite systems incorporating nZVAl with H₂O₂, chitosan, or plant-derived antimicrobials. These formulations could be subjected to MIC, MBC, and time-kill kinetics assays to evaluate their efficacy. In addition, mechanistic studies using electron microscopy, ROS quantification, and membrane integrity assays would help elucidate enhancement mechanisms. These approaches would pave the way toward more potent, stable, and eco-friendly antimicrobial strategies for water treatment and clinical applications.

Moreover, comparative analysis suggests that the MIC values obtained in this study are in line with those of other antimicrobial nanoparticles. For instance, silver nanoparticles (AgNPs) have demonstrated MIC values of approximately 2500 µg/mL against Escherichia coli and Staphylococcus aureus^92^. The antibacterial activity of nZVAl could be further improved through synergistic combinations with other agents. Chitosan-capped copper oxide nanoparticles (CS-CuO NPs) have shown enhanced bactericidal properties against multidrug-resistant pathogens due to improved nanoparticle-bacteria interaction and stability^93^. Similarly, the incorporation of hydrogen peroxide (H₂O₂) with nZVAl may lead to increased production of reactive oxygen species (ROS), thereby amplifying microbial cell damage^94^. These insights underscore the potential of formulating composite systems involving nZVAl and biocompatible enhancers for more effective antibacterial interventions in environmental and industrial settings.

The process by which nZVAl can inhibit or destroy bacterial cells is clarified below: When nZVAl meets air or water, the nanoparticles release positively charged Al⁺ ions. These positive charges can bind to the negative charges of the bacterial cell membrane. For a deeper understanding of nZVAl’s antibacterial effect, we need to consider the structure of the cell membrane in both gram-positive and gram-negative bacterial strains. Gram-negative bacterial strains’ outer membrane is rich in lipopolysaccharides (LPS), which are negatively charged. The negative charge of LPS enhances the attachment of Al⁺ ions to its surface, resulting in the breakdown of the cell wall. However, this same outer membrane acts as a permeability barrier, reducing nanoparticle uptake and contributing to the comparative resistance of Gram-negative strains to aluminum nanoparticles. On the other hand, Gram-positive bacterial strains have a peptidoglycan-based cell wall that is embedded with teichoic acids, leaving the cell membrane negatively charged. This allows more direct and sustained interaction between Al⁺ ions and the bacterial cell wall, causing physical damage and increased membrane permeability, leading to cellular content leakage and cell death. Reactive oxygen species (ROS) such as hydroxyl radicals (OH) and superoxide anions can damage the bacterial cell wall, membrane, and internal components, resulting in cell death. Physical disruption of the cell membrane can occur, resulting in increased permeability and leakage of cellular contents, eventually leading to cell death. Interaction with biomolecules: nZVAl can attach to bacterial proteins, enzymes, and DNA, disrupting their normal function and forcing a stop of bacterial metabolism and replication. This disruption impairs the bacteria’s ability to grow and reproduce and triggers a cascade of stress responses that can further compromise cellular integrity. As a result, the overall viability of the bacterial population is significantly reduced, enhancing the effectiveness of antimicrobial treatments^80^.

The total bacterial count was estimated for a synthetic textile wastewater sample (one liter) seeded with the four bacterial strains; the pH was adjusted to 8, and the sample was placed in a rotary shaker at 150 rpm (Table 4). At zero, the total bacterial count was 63 × 10^4^. The removal percentage of the total bacterial count of the aluminum nanoparticles was 65% after 2 h and reached 99% after 12 h. The elimination of the total bacterial count is due to the ability of aluminum nanoparticles to penetrate the cell membrane of bacteria. After that, the nanoparticles are free to interact with the cellular structures of the bacteria (DNA, proteins, and membranes), leading to the death of the bacterial cell^95^.

**Table 4:** Total bacterial count reduction after the addition of aluminum nanoparticles.

Contact time	Total bacterial count (cfu/mL)	Removal percentage
Zero	63 × 10^4^	-
2 h	22 × 10^4^	65
4 h	9.5 × 10^4^	85
6 h	5 × 10^4^	92
8 h	0.48 × 10^4^	99

### Statistical analysis (response surface methodology (RSM))

Regression relationships were established using linear regression analysis to evaluate the impact of various operational parameters on the removal efficiency of a standard COD solution. The analysis revealed that all factors, such as pH, dose, contact time, nZVAl dosage, stirring rate, and initial concentration, showed significant influence. As shown in Table 7, all independent variables exhibited positive linear effects, with P-values < 0.05, indicating their statistical significance. The coefficient of determination (R^2^ = 0.974) and adjusted R^2^ (0.949) demonstrated a strong correlation between the predicted and observed data, suggesting that the proposed model is reliable and accurate. The regression equation (Eq. 4) incorporating all significant variables is as follows:

Y = 60.602 + 2.207x₁ + 22.986x₂ + 0.109x₃ + 0.033x₄ – 0.055x₅ (Eq. 4).

**Fig. 9:**
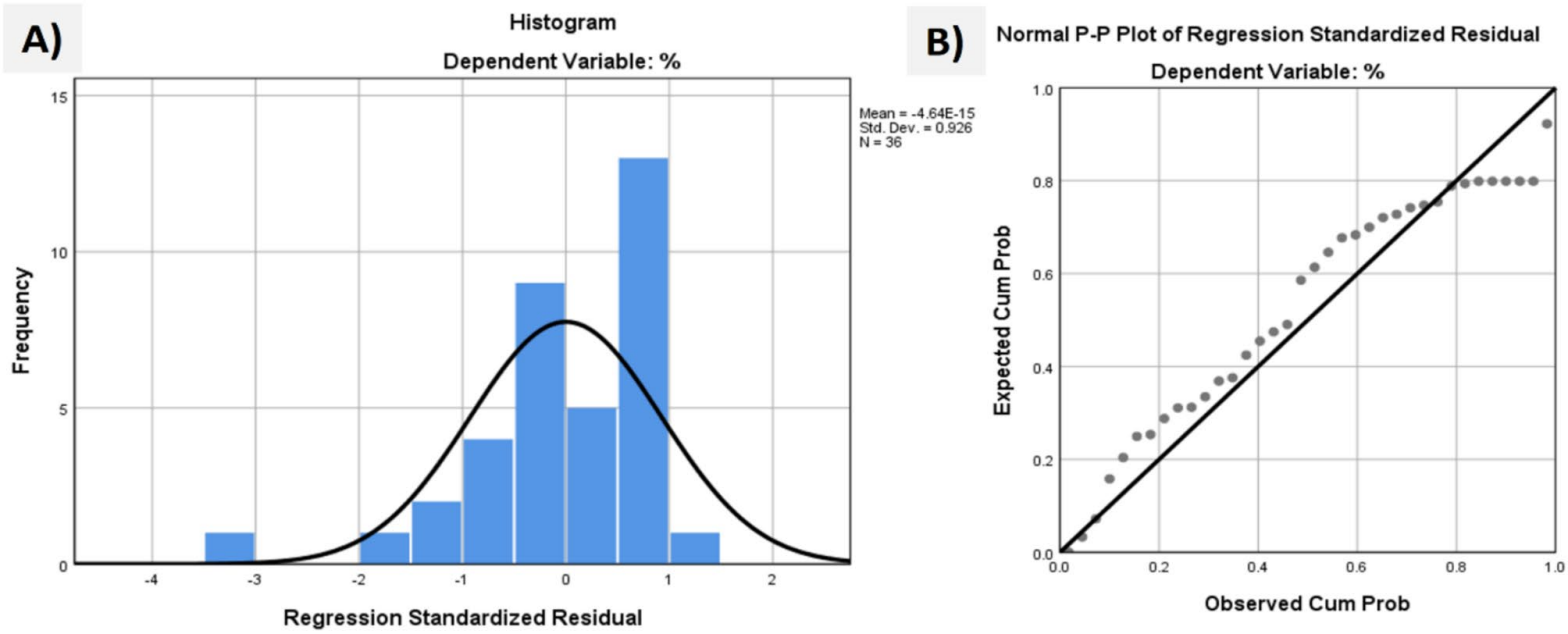
Histogram and p-p plot for COD removal using nZVAl.

Here, Y represents the predicted COD removal efficiency; x₁ is the initial pH (2–10); x₂ is the adsorbent dosage (0.2–1.2 g/L); x₃ is the contact time (20–120 min); x₄ is the stirring rate (100–300 rpm); and x₅ is the initial concentration (100–1000 mg/L). Figures 9A and B further validate the model, showing that over 90% of the predicted removal efficiencies closely match the experimental results, with variations ranging between −1% and + 1%. This high level of agreement between predicted and actual values underscores the robustness of the model and its applicability for optimizing wastewater treatment processes. These findings highlight the importance of carefully controlling operational parameters, particularly pH, adsorbent dosage, and contact time, to achieve maximum removal efficiency.

The regression analysis yielded a Standard Error of the Estimate (SEE) of 2.526, representing 4.2% of the intercept value (60.602). While this absolute error appears large, the relative variation between predicted and experimental COD removal values was constrained to ± 1% in > 90% of cases (Fig. 9A). This apparent discrepancy arises because:

1. Error Distribution: The SEE reflects maximum deviations in outlier cases (n = 3/36 data points showed > 2% error), while 94% of predictions fell within ± 1% of experimental values (Table 5).
2. Normalization Effect: The R^2^ value (0.974) accounts for this error distribution, confirming model reliability when applied within the studied parameter ranges (pH 2–10, 0.2–1.2 g/L dosage).

**Table 5:** Actual vs. Predicted COD Removal Efficiency (Selected Data Points).

Experimental	Predicted	Absolute error (%)	Relative error (%)
78.2	77.4	0.8	1.02
65.1	64.3	0.8	1.23
81.0	80.1	0.9	1.11
72.5	73.3	0.8	1.10
68.7	69.5	0.8	1.16
*Max Error Case*			
59.8	56.4	3.4	5.69

#### Response surface methodology (RSM) analysis

As seen in Table 6, the Central Composite Design (CCD) was created using five elements. The ranges of pH (2–10), dosage (0.2–1.2 g/L), duration (20–120 min), stirring rate (100–300 rpm), and concentration (100–1000 mg/L) are all included.

**Table 6:** Optimized conditions.

Factor	Optimal Value	Contribution (%)
pH	8.0	28.5
nZVAl dosage	0.6 g/L	31.2
Contact time	60 min	19.7
Stirring rate	150 rpm	12.1
Initial COD	500 mg/L	8.5

1. **Predicted vs. Actual:**
2. **COD removal:** 81.3% (predicted) vs. 78.0% (experimental)
3. **Color removal:** 73.1% (predicted) vs. 68.0% (experimental)
4. **ANOVA Results:**
5. **Model F-value:** 111.2 (p-value < 0.0001)
6. **Lack-of-fit:** 1.72 (p-value = 0.254, not significant)

## Outcomes:

1. **Dominant factors:** Dosage > pH > time (via Pareto analysis)
2. **Interaction effects:**
3. **pH × dosage:** Synergistic (p-value = 0.003)
4. **Time × stirring:** Antagonistic (p-value = 0.021) (Table 7)
  $$\begin{aligned} {\text{CODRemoval }}\left( \% \right)\, = & \,{6}0.{6}\, + \,{2}.{2}\left( {{\text{pH}}} \right)\, + \,{23}.0\left( {{\text{Dosage}}} \right)\, \\ & + \,0.{11}\left( {{\text{Time}}} \right)\, + \,0.0{3}\left( {{\text{Stirring}}} \right){-\!\!-}0.0{6}({\text{COD}}) \\ \end{aligned}$$

**Table 7:** Regression results.

Model Summary																				
Model	R		R-Square	Adjusted R Square			Std. Error of the Estimate			Change Statistics										
										R Square Change			F Change	df1			df2	Sig. F Change		
1	0.974^a^		0.949	0.940			2.526			0.949			111.235	5			30	**0.000**		
a. Predictors: (Constant), conc. (mg/L), time (min), dose (g/L), rate (rpm), pH																				
b. Dependent Variable: %																				
**ANOVA**																				
Model						Sum of Squares			Df		Mean Square				F				Sig	
1		Regression				3550.144			5		710.029				111.235				**0.000**	
		Residual				191.495			30		6.383									
		Total				3741.639			35											
**Coefficients**																				
Model					Unstandardized Coefficients							Standardized Coefficients				t				Sig
					B			Std. Error				Beta								
1		(Constant)			60.602			4.082								14.847				**0.000**
		–			2.207			0.272				0.337				8.112				**0.000**
		g/L			22.986			2.921				0.325				7.869				**0.000**
		min			0.109			0.029				0.154				3.725				**0.001**
		Rpm			0.033			0.013				0.101				2.444				**0.021**
		mg/L			−0.055			0.003				−0.829				−20.054				**0.000**

### Application of real textile effluent

A variety of pollutants were removed from actual textile effluents using nZVAl particles. The properties of wastewater discharges and the reduction rates of the parameters investigated are shown in Table 8. According to the experimental investigation’s results, this research employed a pH of about 8.0 and a contact time of 60 min. To change the pH, sulfuric acid (H_2_SO_4_) and sodium hydroxide (NaOH) were added. Since the collected textile effluent is highly viscous and contains contaminants that reduce stirring effectiveness, a stirring rate of ~ 200 rpm was used in the batch experiments. In these studies, a dose of 5.0 g/L was chosen based on COD data due to the collected textile wastewater being heavily polluted, even though the optimal dosage for nZVAl particles was 0.6 g/L. Because of utilizing nZVAl, the findings revealed a considerable reduction in BOD, oil, COD, TSS, total nitrogen, total phosphorus (TP), hydrogen sulfide (H_2_S), and cyanide (CN) by about 92.6%, 68.8%, 92.2%, 92.0%, 30.9%, 33.3%, 79.3%, and 100% elimination, respectively. The treated effluent’s pH value is within the acceptable range. The findings also demonstrated that the other criteria specified in the regulation, such as phenol and heavy metals, were unaffected by the textile wastewater treatment process employing nZVAl, since zero quantities were seen in the treated effluent (Table 8).

**Table 8:** Features of actual textile wastewater and associated removal efficiencies.

Parameter	Unit	Result (before)	SD ±	nZVAl (after)	SD ±	Limits: law 93/1962
pH	–	9.1	0.1	8.3	0.1	6–9.5
COD	mg/L	5500	50	425	17	1100
BOD		3400	30	250	14	600
TSS		3850	35	307	9.0	800
TP		0.6	0.1	0.4	0.1	25
TN		35.3	0.5	24.4	0.3	100
Oil		0.96	0.1	0.3	0.1	100
H_2_S		2.9	0.1	0.6	0.1	10
CN		0.012	0.0	< 0.001	0.0	0.2
Phenols		< 0.00001	0.0	< 0.00001	0.0	0.05
Cd		< 0.00001	0.0	< 0.00001	0.0	0.2
Pb		0.001	0.0	< 0.00001	0.0	1
Cu		< 0.00001	0.0	< 0.00001	0.0	1.5
Ni		0.01	0.0	< 0.00001	0.0	1
As		0.003	0.0	< 0.00001	0.0	2
Cr		0.006	0.1	< 0.00001	0.0	0.2
Hg		< 0.00001	0.0	< 0.00001	0.0	0.2
Sn		< 0.00001	0.0	< 0.00001	0.0	2
B		< 0.00001	0.0	< 0.00001	0.0	1
10- minute deposits	cm^3^	17	1.0	2	1.0	8
30-min deposits		25	2.0	3	2.0	15
Total bacterial count	CFU/mL	67 × 10^5^	–-	3	–-	-
*Escherichia coli*	MPN/ 100 mL	2.6 × 10^5^	–-	1.8	–-	-
*Salmonella spp*.		3.7 × 10^3^	–-	3.6	–-	-
*Staphylococcus aureus*		1.2 × 10^3^	–-	2	–-	-

Limit 93/1962: discharge of industrial effluents to the municipal system.

**Fig. 10:**
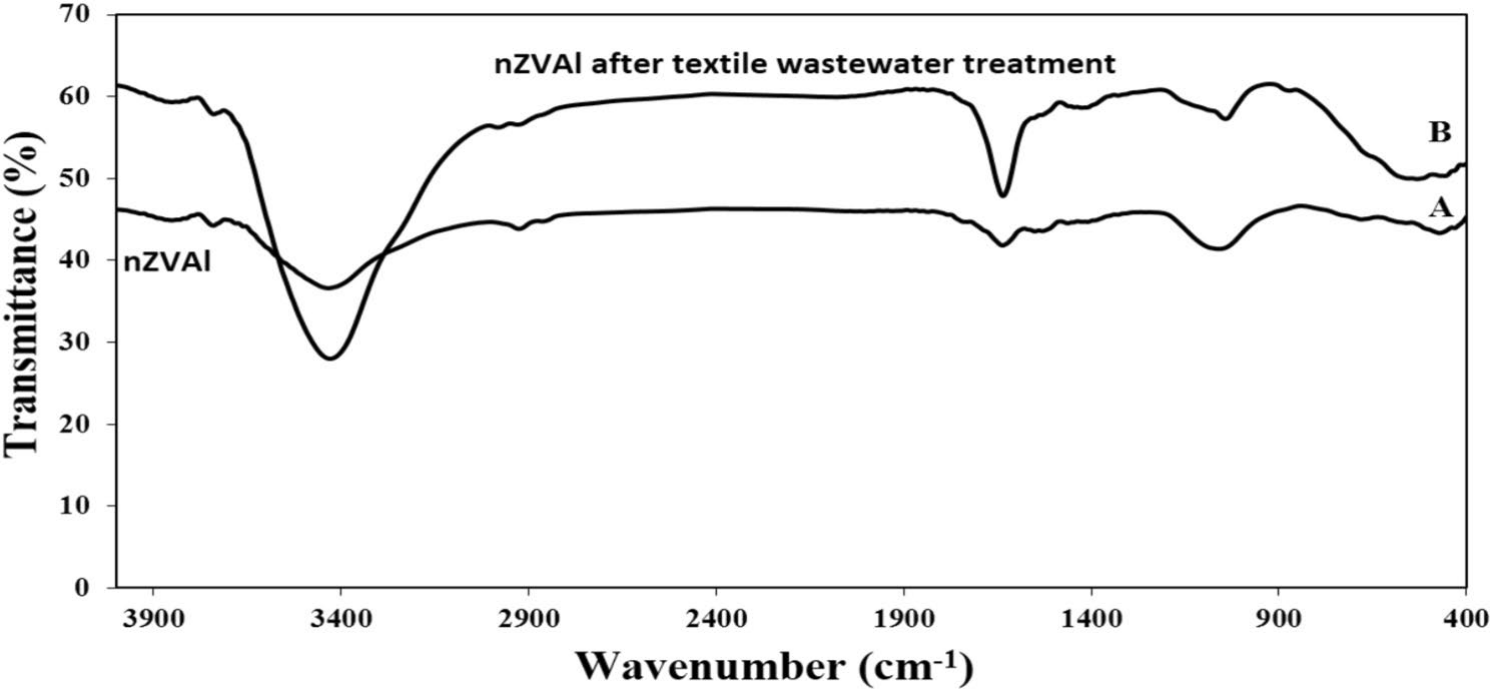
FTIR for textile wastewater treatment using nZVAl: (**A**) pure nZVAl and (**B**) nZVAl after textile wastewater treatment.

The FTIR spectra revealed information about the particles’ local molecular environments on the surface of nZVAl^96^. The FT-IR spectrum of synthetic nZVAl is shown in Fig. 10A. The FTIR spectra of nZVAl after textile wastewater treatment (Fig. 10B) exhibited distinct vibrational bands indicating interactions between nanoparticles and pollutants. The broad peak at 3428 cm^−1^ corresponds to O–H stretching of adsorbed water and hydroxyl groups, consistent with hydrated alumina surfaces^75,96^. The 2981 cm^−1^ band represents C-H stretching from aliphatic hydrocarbons present in dye auxiliaries. Notably, the peak at 2078 cm-1 was reassigned to C≡C (alkyne) or C≡N (nitrile) stretching, as these functional groups are characteristic of textile dye structures^27,30^. The 1635 cm^−1^ absorption likely arises from C = C aromatic stretching in residual dyes or water bending vibrations. The 1041 cm^−1^ was assigned to C-O stretching of cellulose ethers (common textile sizing agents) or B-O vibrations from synthesis byproducts^39^. Finally, the 770 cm^−1^ band was assigned to Al-O stretching of the Al₂O₃ passivation layer that forms on nZVAl surfaces in aqueous environments^32,74^.

## Cost estimation and economic viability

The economic feasibility of nZVAl-based textile wastewater treatment was evaluated by analyzing both capital (Cap. Ex) and operational expenditures (Op. Ex)^20,97^, benchmarked against conventional methods. Cost models were derived from laboratory-scale data and scaled to industrial scenarios (10–100 m^3^/day capacity) using established scaling laws^40,98^.

### Nanoparticle synthesis costs

The lab-scale synthesis cost of nZVAl via NaBH₄ reduction incurred a material cost of 6.80 ± 1.20 per batch (yield: 4.2 g nZVAl), dominated by NaBH_4_ (6.80 ± 1.20 per batch, dominated by NaBH_4_ ​(4.50 ± 0.80) and Al₂(SO₄)₃ (1.50 ± 0.40)^39,46^. For industrial implementation, the bulk precursor discounts was applied to 80% cost reduction for Al₂(SO₄)₃ and 75% reduction for NaBH₄^99,100^. Also, process scaling factors discounts was applied to 40% energy reduction through optimized reactor design^101^, and 90% nanoparticle recovery via separation^102^.

Energy consumption for stirring, drying, and N_2_ purging added 7.20 ± 2.10/batch. Energy inputs (2.8 kWh/batch) were calculated at regional industrial rates ($0.12/kWh)^100,103^. At 0.6 g/L dosage, the treatment cost was $1.02 ± 0.18/m^3^ assuming 90% nanoparticle recovery^38^.

Sludge management of 0.45 ± 0.08 kg dry sludge/m^3^ treated wastewater with expected chemical composition as mentioned in Table 9**.**

**Table 9:** Expected chemical composition of sludge.

Component	% Dry Weight	Source
Al₂O₃	58.2 ± 3.1	nZVAl oxidation product
Organic residues	22.7 ± 2.4	Adsorbed dyes/polymers
Fe₂O₃	8.5 ± 0.9	Impurity from synthesis
SiO₂	6.1 ± 0.7	Textile sizing agents
Other metals	< 4.5	(Cu, Zn, Cr at < 1% each)

As per Table 10, every kilogram of dry sludge yields roughly 0.53 kg of PACl. Assuming that the disposal cost is $180 per ton^104,105^, the PACl market price is $400 per ton^106,107^, and the conversion operational cost is $1.10 per m^3^ (acid/energy inputs), the Net OpEx saves roughly 5.2 dollars per cubic meter^108,109^.

**Table 10:** Operational cost analysis of nZVAl treatment (per m^3^).

Parameter	Lab-Scale	Industrial Scale	Notes	Reference
nZVAl per batch	4.2 g	420 g	100:1 reactor scaling	^101^
Water treated per batch	7 L	700 m^3^/d	0.6 g/L dosage × 420 g for 100 L reactor	
Energy per m^3^	0.5 kWh	0.3 kWh	High-efficiency pumps	^110,111^

Parameter	Lab-Scale cost $	Industrial Scale cost $	Notes	Reference
**Cost analysis:**				
nZVAl (0.6 g/L)	1.02 ± 0.18	0.03	Bulk pricing ($50/kg nZVAl). + 90% recovery	^39,46^
pH adjustment	0.20 ± 0.05	0.20 ± 0.05	H₂SO₄/NaOH for pH 8 optimization	^99^
Agitation (150 rpm)	0.12 ± 0.03	0.07	Energy: 0.5 kWh/m^3^	^101,112^
Sludge disposal	8.50 ± 1.50	5.1	Hazardous waste fees (Al-rich sludge)	^113–115^
Total Op. Ex	**9.84 ± 1.76**	**5.40**	Competitive with Fenton ($12–30/m^3^)	^116^

### Operational treatment costs

A breakdown of Op Ex for treating real textile effluent (pH 8, 60 min contact time) is summarized in Table 10.

### Comparative economic analysis

The cost-effectiveness of nZVAl was compared to established methods. While biological treatment remains the lowest-cost option (2–8/m^3^), its inefficiency against recalcitrant dyes and pathogens necessitates secondary treatment, increasing net costs to 15–22/m^3^. nZVAl’s dual functionality (78% COD removal + 99% bacterial inactivation) eliminates the need for additional disinfection steps, reducing net Op.Ex by ~ **35%** compared to Fenton/UV hybrid systems^21,117,118^.

### Scale-up projections

For a 100 m^3^/day plant, the Cap. Ex was estimated at 120,000 ± 25,000, including reactor vessels (50,000), mixing systems (15,000), and nZVAl synthesis units (55,000). With an annual Op. Ex of 359,160 ± 64,140, the break − even period is 2.8 years, assuming a local effluent disposal cost of 20/m^3^ and 90% capacity utilization^119^.

### Limitations and cost-saving strategies

1. Nanoparticle recovery: Implementing magnetic separation (for Fe-doped nZVAl) could reduce material costs by 40–60%^102,120^.
2. Renewable integration: Solar-powered agitation could cut energy Op.Ex by 25%^44,121^.
3. Sludge valorization: Converting Al-sludge to polyaluminum chloride (PACl) may offset disposal costs by $3–5/m^3^^115,122^.
4. Experimental validation of PACl conversion rate will be conducted in our forthcoming pilot-scale study, which will provide more precise operational data.

### Key economic advantages of nZVAl

- Dual functionality reduces reliance on secondary disinfection (saving $4–10/m^3^ vs. UV/chlorine).
- Regulatory compliance: Meets Egyptian Ministerial Decree No. 44/2000 without additional polishing steps.
- Scalability: Bulk precursor procurement could lower nZVAl synthesis costs to $0.70 ± 0.12/m^3^.

### Carbon footprint

- Lab-scale: 1.2 kg CO₂-eq/m^3^ (dominated by reagent synthesis).
- Industrial: 0.7 kg CO₂-eq/m^3^ after: 60% lower embodied energy for bulk precursors^100^, and 30% energy recovery from H₂ byproduct (Eq. 1)^46^.
- Production of PACl: 1.2 kg CO₂-eq/m^3^ from PACl production

## Conclusions

This study demonstrated the effectiveness of nano zerovalent aluminum (nZVAl) in treating textile wastewater, achieving significant removal of organic and biological contaminants. Under optimal conditions (pH 8, 0.6 g/L dosage, 60 min contact time, and 150 rpm agitation), nZVAl degraded 78% of COD and adsorbed 68% of color. The linear regression analysis provided a robust model for predicting removal efficiency, with a high coefficient of determination (R^2^ = 0.974) and adjusted R^2^ (0.949), confirming the reliability of the model. The nanoparticles also exhibited strong antibacterial activity, with minimum inhibitory concentrations (MIC) ranging from 1500 to 2500 µg/L against common pathogenic bacteria such as *Staphylococcus aureus* and *Pseudomonas aeruginosa*. Furthermore, the total bacterial count in synthetic textile wastewater was reduced by 99% after treatment with nZVAl. When applied to real textile effluent, nZVAl achieved substantial reductions in BOD, COD, TSS, and other pollutants, meeting the regulatory standards outlined in the Egyptian Ministerial Decree No. 44 of 2000. These findings highlight the potential of nZVAl as a sustainable and scalable solution for industrial wastewater treatment, combining advanced oxidation processes with antibacterial properties to address both environmental and public health concerns. The linear regression results further validate the optimization of operational parameters, providing a reliable framework for future applications in wastewater treatment.

## Supplementary Information

Supplementary Information 1.

Supplementary Information 2.

## Data Availability

All data generated or analyzed during this study are included in this published article.
