# Supplementary figures and images for: Sustainable textile wastewater remediation using nano zerovalent aluminum for organic removal and pathogen inactivation
Source: Sci Rep. 2025 Oct 29;15:37784. doi: 10.1038/s41598-025-21563-9 (PMC12572131; doi:10.1038/s41598-025-21563-9)

**Figure S1. Flow Chart**


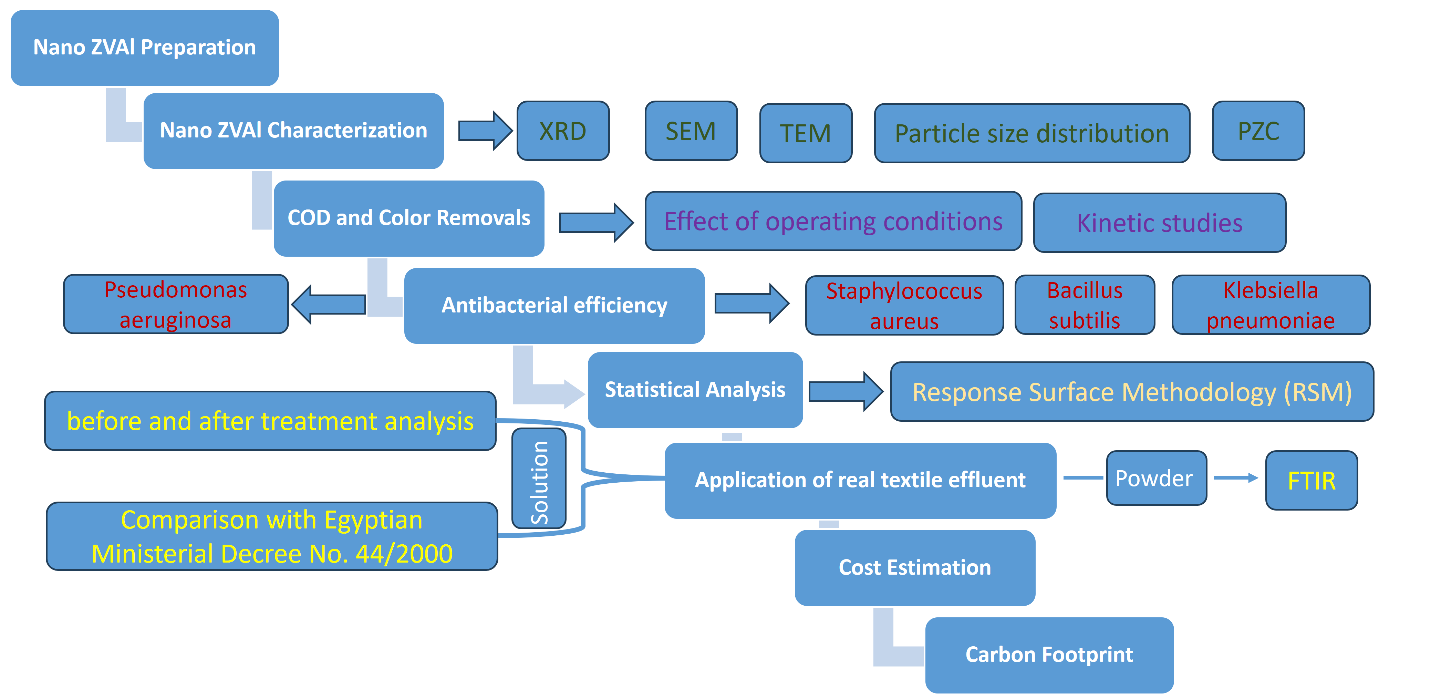

Supplement: Supplementary file 1 — Supplementary Information 1. [file 41598_2025_21563_MOESM1_ESM.docx]
