# Supplementary material for: Sustainable textile wastewater remediation using nano zerovalent aluminum for organic removal and pathogen inactivation
Source: Sci Rep. 2025 Oct 29;15:37784. doi: 10.1038/s41598-025-21563-9 (PMC12572131; doi:10.1038/s41598-025-21563-9)
Supplement: Supplementary file 2 — Supplementary Information 2. [file 41598_2025_21563_MOESM2_ESM.docx]

**Table S1. Chemicals and Reagents**

| Chemical Name | Purity | CAS Number | Supplier | Purpose |
| --- | --- | --- | --- | --- |
| Aluminum sulfate octadecahydrate (Al₂(SO₄)₃·18H₂O) | ≥98% | 7784-31-8 | Sigma-Aldrich | nZVAl precursor |
| Sodium borohydride (NaBH₄) | ≥96% | 16940-66-2 | Merck | Reducing agent |
| Potassium chloride (KCl) | ≥99.5% | 7447-40-7 | Fisher Scientific | PZC determination |
| Sulfuric acid (H₂SO₄) | 95-98% | 7664-93-9 | Loba Chemie | pH adjustment |
| Sodium hydroxide (NaOH) | ≥97% | 1310-73-2 | Sigma-Aldrich | pH adjustment |
| Ethanol (C₂H₅OH) | ≥99.8% | 64-17-5 | Carlo Erba | Co-solvent |
| Ciprofloxacin | USP grade | 85721-33-1 | Fluka | Antibacterial control |
| Mueller-Hinton agar | N/A | 9002-18-0 | Oxoid | Microbial assays |

**Table S2. Nonlinear equations of kinetic models.**

| Kinetic model name | Description | Equations | Reference |
| --- | --- | --- | --- |
| Pseudo First Order | Suggests physisorption dominance, driven by weak interactions such as hydrogen bonding and van der Waals forces. | $Q_{t}= Q_{e}(1-e^{(-K_{1}t})$ | [1-8]. |
| Pseudo Second Order | Indicates chemisorption, where covalent electron exchange/sharing occurs between sorbate and sorbent. | $Q_{t}= \frac{Q_{e}^{2}K_{2}t}{(1+Q_{e}K_{2}t)}$ |  |
| Elovich | Describes chemically bonded adsorption on heterogeneous surfaces, originally for gas-phase systems but extended to aqueous-phase contaminant transfer | $Q_{t}= \frac{1}{\beta}Ln(\alpha\beta_{t})$ |  |
| Avrami | Adapts crystallization kinetics to adsorption, accounting for phase transformations (e.g., solid-state reactions) with variable reaction dimensionality. | $Q_{t}= Q_{e}(1-e^{(-K_{av}t^{n_{av}}})$ |  |
| Intraparticle model | Attributes adsorption to pore diffusion within the adsorbent (e.g., GT-nZVI), ignoring film diffusion but incorporating internal mass transfer resistance | $Q_{t}= K_{id}t^{0.5}+C_{i}$ |  |

**Table S3. Results of experimental and calculated Qt (mg/g) for COD removal using nZVAl.**

| Material | Exp. Qt | Calc. P.F.O Qt | Calc. P.S.O Qt | Calc. Elovich Qt | Calc. Avrami Qt | Calc. Intraparticle Qt |
| --- | --- | --- | --- | --- | --- | --- |
| nZVAl | **575** | 564.4 | 566.1 | 572.7 | **572.3** | 579.2 |
|  | **600** | 639.7 | 621.4 | 611.9 | **612.3** | 607.8 |
|  | **650** | 649.8 | 642.4 | 634.8 | **635.3** | 629.7 |
|  | **650** | 651.1 | 653.4 | 651.1 | **651.3** | 648.1 |
|  | **658** | 651.3 | 660.2 | 663.7 | **663.6** | 664.4 |
|  | **675** | 651.3 | 664.8 | 674.0 | **673.5** | 679.1 |

References

1. Zeldowitsch, J., *Über den mechanismus der katalytischen oxydation von CO an MnO2.* Acta Physicochim. URSS, 1934. **1**: p. 364-449.

2. Avrami, M., *Kinetics of phase change. II transformation‐time relations for random distribution of nuclei.* The Journal of chemical physics, 1940. **8**(2): p. 212-224.

3. Ho, Y.-S. and G. McKay, *Kinetic models for the sorption of dye from aqueous solution by wood.* Process Safety and Environmental Protection, 1998. **76**(2): p. 183-191.

4. Ho, Y.-S. and G. McKay, *Pseudo-second order model for sorption processes.* Process biochemistry, 1999. **34**(5): p. 451-465.

5. Fola, A.T., A.A. Idowu, and A. Adetutu, *Removal of Cu2+ from aqueous solution by adsorption onto quail eggshell: Kinetic and isothermal studies.* Journal of Environment & Biotechnology Research, 2016. **5**(1): p. 1-9.

6. Mahmoud, A.S., et al., *Effective chromium adsorption from aqueous solutions and tannery wastewater using bimetallic Fe/Cu nanoparticles: response surface methodology and artificial neural network.* 2021. **14**: p. 11786221211028162.

7. Mahmoud, M. and A.S.J.E.M. Mahmoud, *Wastewater treatment using nano bimetallic iron/copper, adsorption isotherm, kinetic studies, and artificial intelligence neural networks.* 2021. **4**: p. 1455-1463.

8. Rabie S. Farag, M.M.E., Ahmed S. Mahmoud, Mohamed K. Mostafa, Ahmed Karam, and R.W. Peters. *Study the Degradation and Adsorption Processes of Organic Matters from Domestic Wastewater using Chemically Prepared and Green Synthesized Nano Zero-Valent Iron*. in *2019 Annual AIChE Meeting; Orlando, FL, November 10 - 15, 2019*. 2019. <https://www.researchgate.net/publication> ….
